# Flexible Obturator With a Full Digital Workflow: A Proof-of-Concept Case Report

**DOI:** 10.7759/cureus.98262

**Published:** 2025-12-01

**Authors:** Orianne Borel, Dov Derman, Geraldine Lescaille, Benjamin Pomes

**Affiliations:** 1 Odontology Department, Health Faculty, Université Paris Cité, Paris, FRA; 2 Odontology Department, Assistance Publique-Hôpitaux de Paris (AP-HP) Pitié-Salpêtrière Hospital, Paris, FRA

**Keywords:** cad-cam, computed tomography (ct ), maxillary defect, maxillectomy rehabilitation, maxillo-facial prosthesis, obturator prosthesis, three-dimensional (3d) printing

## Abstract

Oral cancer treatment often results in maxillary defects that require prosthetic rehabilitation to restore function and aesthetics. Palatal obturators, particularly flexible silicone obturators, are commonly used but are difficult to design and fabricate in patients with post-radiation trismus or extensive defects.

This case report presents an innovative, fully digital workflow from design to manufacture of a self-retaining flexible palatal obturator.

Using cone-beam computed tomography (CBCT) scans and segmentation software, a three-dimensional (3D) model of the defect was created, which was then used to design a hollow obturator. The obturator was 3D-printed with flexible IBT (indirect bonding tray) resin (Formlabs, Somerville, MA).

The 3D-printed flexible obturator was clinically validated chairside. A qualitative assessment was performed by the patient and showed comparable functional results between the digital obturator and conventional obturator, with notable advantages in weight reduction.

This innovative protocol bypasses the traditional challenges of impression taking, offering a reproducible and accessible method for the prosthetic management of complex maxillary defects.

## Introduction

Oral cancer, referring to cancers of the oral cavity and oropharynx, ranks as the sixth most prevalent cancer worldwide [1]. Surgical resection remains the primary treatment, often combined with chemotherapy and postoperative radiotherapy [2]. These treatments can result in complications such as trismus and maxillary defects, leading to oronasal communication and impaired mastication, speech, and aesthetics [3].

Rigid resin palatal obturators are dental prostheses designed to close maxillary defects and restore oral function [4]. Their insertion and removal remain critical, particularly in patients with post-radiotherapy trismus [5]. An optimal solution may be a removable dental prosthesis with a dissociated flexible silicone palatal obturator to allow insertion of the dental prosthesis in two parts. The self-retaining flexible silicone obturator can also contribute to the stability and retention of the prosthesis [6].

The design of these prostheses has its limitations, particularly the physical impression. This step is difficult for the patient, with the placement of materials in contact with fragile internal anatomical structures, and challenging for the practitioner, confronted with several technical difficulties, such as limited mouth opening [7]. To overcome these limitations, computer-aided design and manufacturing (CAD/CAM) techniques have been explored.

In recent years, CAD/CAM technologies have been increasingly applied in dentistry, allowing clinicians to digitize several steps of the workflow. These include three-dimensional (3D) scanning, digital modelling, and additive manufacturing (3D printing).

Several recent studies have demonstrated the feasibility of designing obturators through digital workflows; however, all such devices have been fabricated from rigid resin materials, limiting their adaptability for specific clinical situations like small or tooth-supported defects [8-14].

The objective of this work was to develop and clinically validate a self-retentive flexible obturator produced through a fully digital workflow. This proof of concept aims to extend the use of digital manufacturing techniques to flexible obturators, enabling the creation of prostheses that are both functional and comfortable for patients with extensive maxillary defects.

## Case presentation

**Figure 1:**
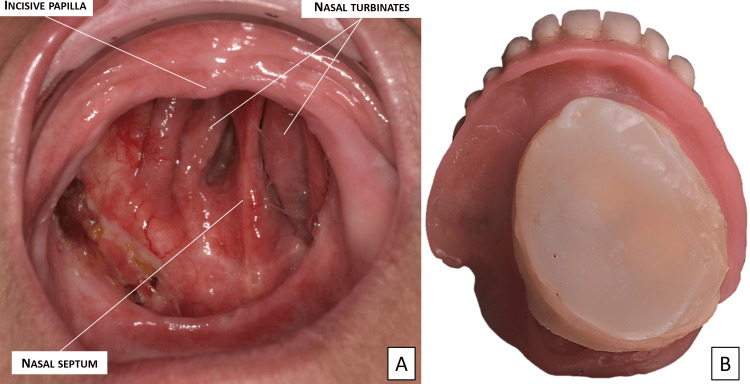
Clinical exam. (A) Class 2 type D palatal defect with bucco-naso-sinusal communication. (B) Patient’s damaged soft silicone obturator and full removable prosthesis.

The patient is a 78-year-old man with a history of cavum cancer diagnosed in 2004 and treated with resective surgery combined with chemotherapy and radiotherapy. The patient presented a class 2 type D palatal defect (modified Brown classification, 2010) [15] with bucco-naso-sinusal communication (Figure 1A) and has had a flexible silicone obturator since 2005. His palatal obturator was damaged. It could no longer seal the maxillary defect and required to be changed (Figure 1B).

We decided to implement a fully digital protocol for the design and manufacture of the new palatal obturator.

Digital recording of the defect

The acquisition of the defect was based on a CBCT scan (Planmeca CBCT system; Planmeca, Helsinki, Finland) of the maxillofacial region.

Segmentation

**Figure 2:**
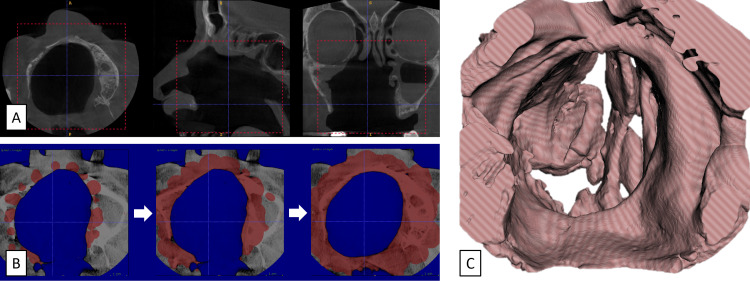
Segmentation steps. (A) Selection of area of interest (DICOM file). (B) Segmentation progress. (C) 3D model (STL file). 3D, three-dimensional.

The 2D DICOM files were converted to 3D STL format by segmentation (ITK snap 3.8.0 software; Penn Image Computing and Science Laboratory, University of Pennsylvania, Philadelphia, PA) (Figure 2). The minimum threshold at which tissues were segmented is chosen to segment the tissues of interest. It was set at -350 HU, close to the values for the lung, an organ that also includes soft tissues and air cavities. Segmentation bubbles were then added to initiate automatic segmentation. The more the segmentation bubbles, the more fast and accurate the segmentation is. For point evolution, strength and smoothness have been left at normative values (1 and 0.200). The 3D model was then displayed by pressing the "update" button and exported in STL format.

Computer-aided design

**Figure 3:**
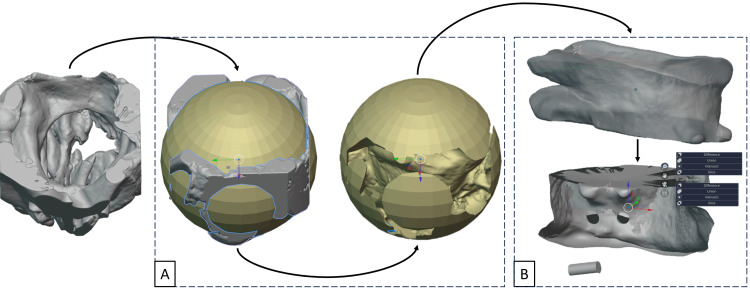
Final obturator design. (A) Boolean subtraction. (B) Design of the final palatal obturator.

The modelling step was performed using Blender software (Blender Foundation, Amsterdam, Netherlands), which produced a 3D model of the obturator derived from the 3D model of the defect. Boolean subtraction, which consists of subtracting the 3D model of the defect from a reference volume, was used to obtain a positive model from the negative model of the defect (Figure 3A). This positive model served as the basis for creating the obturator. The subtraction product was cleaned up by removing areas that were not attached and that would not be used by the obturator. "Smooth" and "Sculpt" tools made the obturator's contours softer and less aggressive. The obturator was then hollowed out at its centre, with a wall thickness of around 1.5 mm. Two wells were created to allow uncured resin to escape during 3D printing (Figure 3B). The resulting digital file can then be used to 3D print the obturator.

Computer-aided manufacturing: Stereolithography and IBT resin

**Figure 4:**
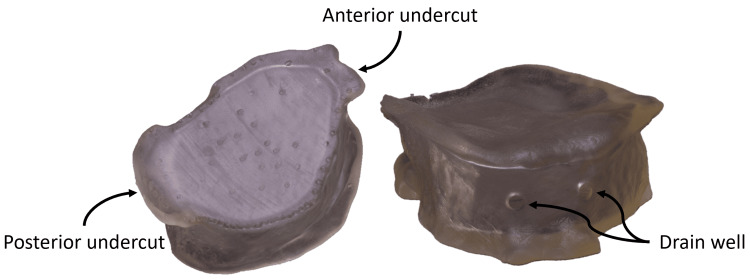
Final hollow obturator after 3D printing. 3D, three-dimensional.

Three-dimensional printing of the obturator was carried out using stereolithography (Formlab 3D printer) and a flexible, translucent IBT (indirect bonding tray) resin (FormLabs, Somerville, MA) (Figure 4).

IBT resin is a Class I biocompatible photopolymer typically used for intraoral bonding trays. It is translucent and elastic, making it suitable for validating a clinical proof-of-concept with an armchair fitting of the 3D-printed obturator.

Clinical validation of the 3D-printed hollow obturator

**Figure 5:**
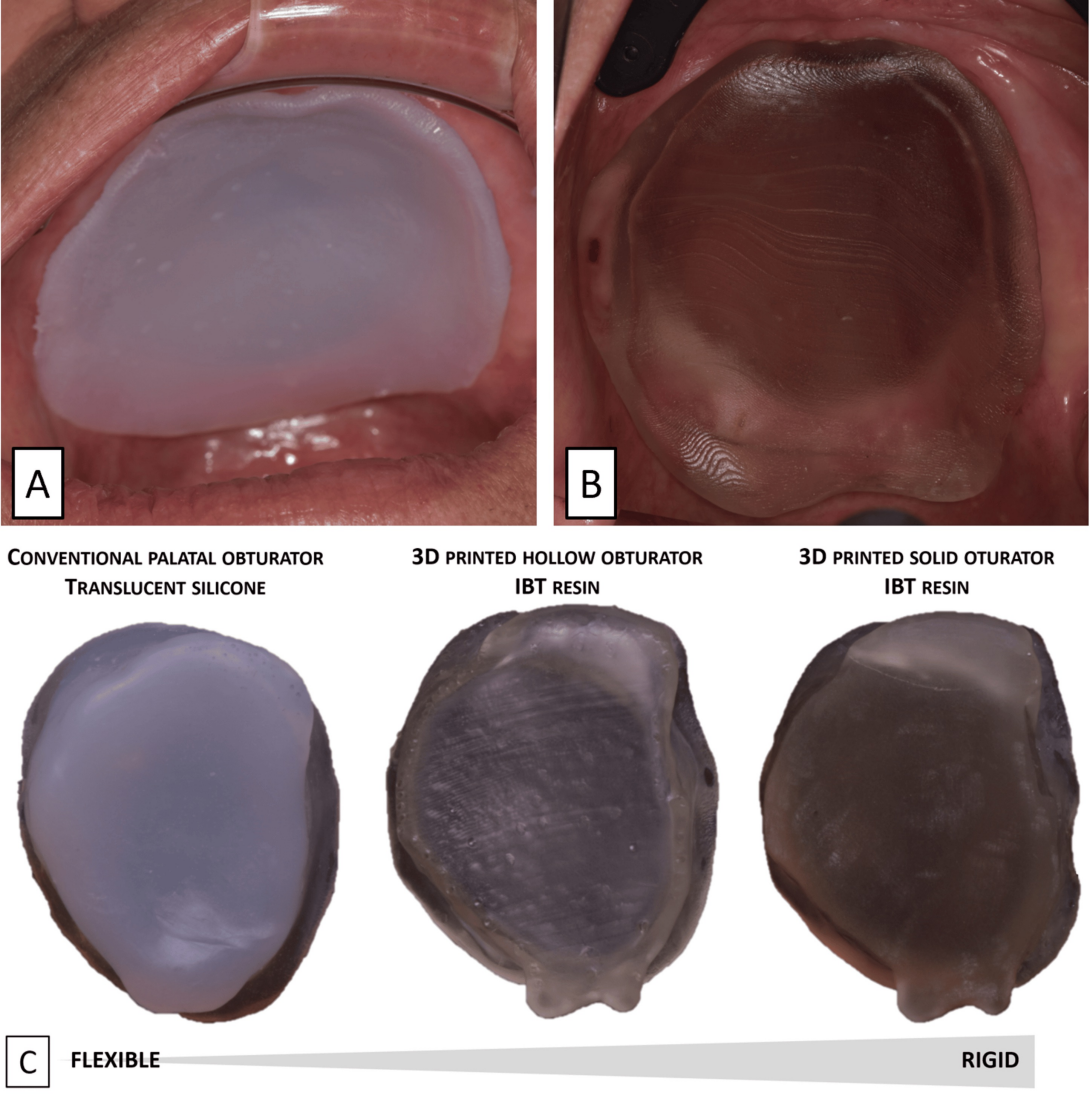
Clinical validation of the 3D-printed obturator. (A) Conventional silicone obturator placed in the defect. (B) 3D-printed obturator placed in the defect. (C) Comparison of traditional and 3D-printed palatal obturators. IBT, indirect bonding tray; 3D, three-dimensional.

The clinical validation of the 3D-printed hollow obturator demonstrated promising results. After minor adjustments to the pronounced front undercut in the design, the obturator was successfully inserted into the defect (Figure 5B). For comparison, a flexible silicone obturator was fabricated using an alginate physical impression (Figure 5A). Both obturators showed comparable performance in terms of adaptation to the defect boundaries, stability, tightness during swallowing, and phonation. However, the patient noted that the 3D-printed hollow obturator was lighter, offering greater comfort and stability. While the hollowing process increased flexibility (Figure 5C), the insertion of the 3D-printed obturator required more care due to the lower flexibility of the IBT resin compared to silicone. 

## Discussion

The present work introduces a clinical proof-of-concept demonstrating the feasibility of producing an obturator through an entirely digital workflow, from the acquisition of the defect to the 3D printing of a self-retaining flexible obturator.

This innovative method bypasses the difficulties associated with impression taking and could be applied to patients with significant mouth-opening limitations.

While digital obturator fabrication has been previously explored, existing studies have focused exclusively on rigid resin obturators [8-14]. To our knowledge, this is the first documented case in which a flexible, self-retentive obturator has been designed and manufactured using a fully digital process.

This innovation extends the field of digital maxillofacial prosthetics beyond rigid materials, confirming the feasibility of adapting digital manufacturing to flexible structures. From a clinical perspective, flexible obturators are particularly useful for patients with complete edentulism or severe trismus, where the elasticity of the material allows deformation during insertion and self-retention once in place [16]. This property facilitates insertion despite limited mouth opening and helps stabilize complete removable prostheses.

In this case, both obturators, conventional and digital, were evaluated on the same patient. The functional performance of the digital obturator was comparable to that of the conventional silicone one in terms of adaptation, sealing, and phonation, as verified during swallowing tests with water. The patient reported enhanced comfort and a lighter sensation when wearing the digital obturator. These qualitative results suggest that a fully digital workflow could offer a viable alternative to conventional fabrication.

Nevertheless, this study remains a single-patient proof-of-concept, and its conclusions are therefore limited. Quantitative assessment methods would be necessary in future work to confirm these preliminary findings.

Moreover, because the flexible resin used in this case (IBT, Formlabs) has Class I biocompatibility, its intraoral use was limited to chairside adaptation. Future developments with Class IIa biocompatible flexible materials will be essential to enable long-term clinical use and follow-up evaluation.

This proof of concept thus provides an important foundation for future clinical applications, showing that digital technologies can successfully be extended to the fabrication of flexible, self-retentive obturators, combining precision, comfort, and adaptability - an advancement that had not been validated in a clinical setting until now.

## Conclusions

The new all-digital protocol for the self-retaining flexible obturator developed in this article achieved a proof of concept.

Compared with a conventionally fabricated silicone obturator, the digitally manufactured flexible obturator provided similar functional results while offering a lighter weight and improved comfort. This original and novel approach opens new prospects for the prosthetic treatment of compromised maxillary defects. By avoiding the physical impression, which is the most crucial and difficult part for both patient and dentist, this technique makes the manufacture of flexible obturators more accessible and reproducible.
